# Influencing Factors and Applicability of the Viability EMA-qPCR for a Detection and Quantification of *Campylobacter* Cells from Water Samples

**DOI:** 10.1371/journal.pone.0113812

**Published:** 2014-11-20

**Authors:** Diana Seinige, Maren von Köckritz-Blickwede, Carsten Krischek, Günter Klein, Corinna Kehrenberg

**Affiliations:** 1 Institute of Food Quality and Food Safety, University of Veterinary Medicine Hannover, Foundation, Hannover, Germany; 2 Institute for Physiological Chemistry, University of Veterinary Medicine Hannover, Foundation, Hannover, Germany; Charité-University Medicine Berlin, Germany

## Abstract

In recent years, increasing numbers of human campylobacteriosis cases caused by contaminated water have been reported. As the culture-based detection of *Campylobacter* is time consuming and can yield false-negative results, the suitability of a quantitative real-time PCR method in combination with an ethidium monoazide pretreatment of samples (EMA-qPCR) for the rapid, quantitative detection of viable *Campylobacter* cells from water samples was investigated. EMA-qPCR has been shown to be a promising rapid method for the detection of viable *Campylobacter* spp. from food samples. Application of membrane filtration and centrifugation, two methods frequently used for the isolation of bacteria from water, revealed a mean loss of up to 1.08 log_10_ cells/ml from spiked samples. Both methods used alone lead to a loss of dead bacteria and accumulation of viable bacteria in the sample as shown by fluorescence microscopy. After filtration of samples, no significant differences could be detected in subsequent qPCR experiments with and without EMA pretreatment compared to culture-based enumeration. High correlations (R^2^ = 0.942 without EMA, R^2^ = 0.893 with EMA) were obtained. After centrifugation of samples, qPCR results overestimated *Campylobacter* counts, whereas results from both EMA-qPCR and the reference method were comparable. As up to 81.59% of nonviable cells were detected in pond water, EMA-qPCR failed to detect correct quantities of viable cells. However, analyses of spiked tap water samples revealed a high correlation (R^2^ = 0.863) between results from EMA-qPCR and the reference method. After membrane filtration, EMA-qPCR was successfully applied to *Campylobacter* field isolates, and results indicated an advantage over qPCR by analysing defined mixtures of viable and nonviable cells. In conclusion, EMA-qPCR is a suitable method to detect viable *Campylobacter* from water samples, but the isolation technique and the type/quality of the water sample impact the results.

## Introduction

Campylobacteriosis remains the most commonly reported bacterial foodborne disease in humans worldwide [1]. The incidence of campylobacteriosis has risen recently, with more than 200,000 confirmed cases in the European Union reported each year [2], [3]. *Campylobacter* (*C.*) infections are clinically manifested by diarrhoea, fever, and abdominal cramps, and, in certain cases, may be followed by long-term sequelae such as Guillain-Barré syndrome or reactive arthritis [3]. Although it is generally agreed that the major source of human infections is contaminated poultry meat, there is evidence that surface and/or drinking water also acts as a vehicle for *C. jejuni* and *C. coli* transmissions to humans [4], [5], [6], [7], [8]. Rivers or other natural aquatic environments can be contaminated with thermotolerant *Campylobacter* by raw sewage, discharge from wastewater-treated agricultural land, or faeces from wild or domestic animals [9], [10], [11], [12], [13].

Assessments of microbiological water quality generally focus on testing for indicator bacteria, like *Escherichia coli*, which are used to estimate the exposure of drinking water to faecal contamination [12], [14]. Nevertheless, the onset of human *Campylobacter* infections or outbreaks may necessitate the inclusion of thermotolerant *Campylobacter* in microbial water analyses [4], [15]. Water analysis laboratories often use the ISO standard method 17995:2005 [16], which initially includes a filtration step of the water samples, followed by bacterial enrichment and cultivation on selective agar plates. However, the ISO method is time consuming and often fails to detect *Campylobacter* from water samples [17]. This may be the result of higher amounts of injured or viable but nonculturable (VBNC) cells under stressful environmental conditions, as is the case in water [17]. To circumvent the limitations of culture-based methods, quantitative real-time PCR (qPCR) approaches for the detection of *Campylobacter* from water have been developed and successfully applied [18], [19], [20], [21]. However, the lack of differentiation between DNA from viable and nonviable cells restricts the implementation of these PCR-based techniques for routine diagnostic applications [21]. Recently, a sample pretreatment with intercalating dyes like ethidium monoazide (EMA) or propidium monoazide (PMA) was proposed to address this problem. These dyes cross the membranes of damaged cells, covalently bind to DNA after photoactivation, and, thus, block PCR amplification of DNA from nonviable cells [22]. EMA- or PMA-qPCR assays have been successfully used to quantify *Campylobacter* from poultry products [22], [23], [24] but little is known about the applicability of a viability qPCR to determine the quantities of *Campylobacter* from water samples [25], [26].

Therefore, the objectives of this study were to assess the general suitability of an EMA-qPCR method for the quantification of *Campylobacter* cells from water samples. For this purpose, two methods for the recovery of cells from water samples were comparatively analysed, and live/dead ratios after inoculation of cells in different type of water were considered.

## Materials and Methods

### Bacterial strains and culture conditions

The type strain *C. jejuni* DSM 4688^T^ (Leibniz-Institut, Deutsche Sammlung von Mikroorganismen und Zellkulturen, Braunschweig, Germany), *C. jejuni* (n = 7), and *C. coli* (n = 6) field isolates of avian origin, representing part of the bacterial strain collection of the Institute of Food Quality and Food Safety, were used for the experiments. Bacterial strains were stored at −80°C and cultivated on charcoal cefoperazone desoxycholate agar plates (Qxoid, Wesel, Germany) at 41.5±1°C for 48 h under microaerobic conditions (5% O_2_, 10% CO_2_, 85% N_2_). For the culture-based enumeration of cells, 10-fold serial dilutions from 10^−1^ to 10^−6^ were prepared from bacterial suspensions. For each dilution, aliquots of 0.1 ml were spread on at least 10 agar plates. Colonies were counted after 48 h of incubation from agar plates containing 1 to 300 colonies. For the inactivation of *Campylobacter*, cells were heat-killed at 70°C for 15 min in a water bath as previously described [24].

### Recovery of *Campylobacter* cells from water samples

To concentrate the *Campylobacter* cells from water samples, two different isolation methods were compared: the microfilter technique and the low-rotation centrifugation method. For the microfilter technique, water samples (10 ml) were spiked with varying concentrations (CFU/ml) of *Campylobacter* and filtrated through a cellulose ester membrane filter (Biosart 100 Monitore, 0.45-µm pore size, Sartorius, Göttingen, Germany) using a vacuum pump (Typ MP 1 Pfeiffer, Asslar, Germany). After filtration, membrane filters were carefully removed with sterile forceps, and bacteria were rinsed off the filters using 10 ml of 0.9% NaCl. Subsequently, the filter was put into an Erlenmeyer flask containing the rinsing fluid, and the flask was shaken for 2 min on a vortex mixer to remove bacteria adhered to the membrane. For low-rotation centrifugation, 100-ml samples, spiked with varying concentrations (CFU/ml) of *Campylobacter*, were centrifuged for 15 min at 5141× *g* in Falcon tubes. The supernatant was discarded, and bacterial pellets were resuspended in 0.9% NaCl to a total volume of 1 ml. All experiments were performed with two different types of water. Pond water was collected from a water pond in Isernhagen, Germany, and tap water was collected from the public water supply in Hannover, Germany. For control purposes, 0.9% NaCl was included. A chemical analysis of a pond water sample was commissioned (Umwelt Analyse Zentrum, Filderstadt, Germany), and data from the chemical water analyses of tap water were provided from municipal utilities (Stadtwerke Hannover, Germany). In addition, a microbiological water analysis was performed in the laboratory for water analyses of the University of Veterinary Medicine Hannover, Foundation, Germany. To test a more varied selection of water types, rain water (collected in Hannover, Germany) and commercially available bottled still mineral water (EDEKA, Hamburg, Germany) were included in the EMA-qPCR experiments. The water samples were spiked with various amounts of viable or mixed (1:10 and 1:100, live/dead ratios) *Campylobacter* cells as required. Three independent experiments were performed.

### DNA isolation, qPCR, and EMA-qPCR

After filtration or centrifugation of the spiked water samples, EMA treatment was carried out as previously described for the detection of *Campylobacter* from poultry products [24]. In brief, the intercalating dye EMA (Invitrogen, Darmstadt, Germany) was added to samples at a final concentration of 10 µg/ml. After a 15-min incubation in the dark, tubes were placed in an ice-cooled box and subjected to photoactivation for 15 min using a 500-W halogen lamp source (düwi type R7s, REV Ritter GmbH, Mömbris, Germany). DNA was extracted from 1 ml of EMA-treated or untreated samples using the Qiagen DNeasy Blood and Tissue Kit (Qiagen, Hilden, Germany) according to the manufacturer's recommendations. The qPCR was performed with the SureFood PATHOGEN *Campylobacter* PLUS LC kit (Congen, Berlin, Germany) according to the manufacturer's instructions. The detection limit for this kit is less than or equal to five copies/sample. PCR conditions were 60 s at 95°C, followed by 45 cycles of 95°C for 10 s and 60°C for 15 s. For qPCR analysis, a LightCycler 480 II instrument (Roche Diagnostics, Mannheim, Germany) was used. For EMA-qPCR experiments, an internal standard of EMA-pretreated cells was used along with an adapted standard curve to compensate for the C_t_ shift caused by the pretreatment of cells with the intercalating dye [24]. All experiments were carried out three times.

### Fluorescence microscopy

To determine changes in the membrane integrity of *Campylobacter* cells as a consequence of the method selected to concentrate the cells or due to the incubation of cells in different water types, fluorescence microscopy was used. For this, a 1:1 mixture of the following nucleic acid dyes was added to 1 ml of the samples: SYTO 9 as an indicator of viable bacteria and propidium iodide as an indicator of dead bacteria (LIVE/DEAD *Bac*Light Bacterial Viability Kit, Invitrogen, Darmstadt, Germany). The suspensions were incubated in the dark for 10 min, followed by a 5-min centrifugation step at 3000 rpm. Microscopic evaluation was carried out using a Leica DMI6000CS confocal microscope (Leica Microsystems, Wetzlar, Germany) in combination with a HCX PL APO lambda blue 63.0×1.40 oil UV objective. Settings were adjusted in accordance with control preparations using heat-killed bacteria (15 min, 70°C). A minimum of six randomly selected images were acquired per individual sample and used for quantification of green (live), red (dead), and orange (intermediate) bacteria. Experiments were carried out at three independent times.

### Statistical analyses

Statistical analyses were performed using GraphPad Prism 6 Software (San Diego, USA). The results from microbiological cell enumeration and either qPCR or EMA-qPCR, and results from fluorescence microscopy were statistically analysed using one-way ANOVA with independent variables as the isolation method (membrane filtration, centrifugation) or water type (pond water, tap water). If the F-statistic was significant (*p*<0.05), the Dunett's multiple comparison post-hoc test or Tukey's multiple comparison post-hoc test for repeated measurements was subsequently applied, considering a significance level of 0.05. To assess the relationship between culture-based enumeration and qPCR results, a Pearson correlation analysis was performed. For method comparisons, the Bland-Altman analysis was used.

## Results

### Comparison of the filtration and centrifugation methods for the recovery of *Campylobacter* cells

To assess the suitability of the centrifugation and membrane filtration techniques for the concentration of cells prior to qPCR analyses with and without EMA, a saline solution was inoculated with 10^7^ CFU/ml viable *C. jejuni* DSM 4688^T^ cells and diluted in a 10-fold dilution series up to 10^−6^ CFU/ml. NaCl at a concentration of 0.9% was used as a solvent to exclude a cell-compromising effect of the water through osmotic pressure. Microbiological cell enumeration was done in triplicate before and after centrifugation or filtration of the spiked NaCl suspensions (dilution steps 10^−4^, 10^−5^, and 10^−6^) to determine the mean loss of cells. A mean significant (*p*<0.05) reduction of 1.08±0.021 SD log_10_ CFU/ml *C. jejuni* was detected after centrifugation, whereas the mean reduction after membrane filtration was 0.73±0.161 SD log_10_ CFU/ml (*p*<0.05) (Figure S1). However, the number of lost cells was independent of the quantities of *Campylobacter* cells in the spiked samples. In total, the mean loss of bacteria was significantly (*p*<0.05) higher after centrifugation of *C. jejuni* cells compared to membrane filtration.

### Concentrating cells from liquid samples affects the proportions of viable and nonviable cells

**Figure 1:**
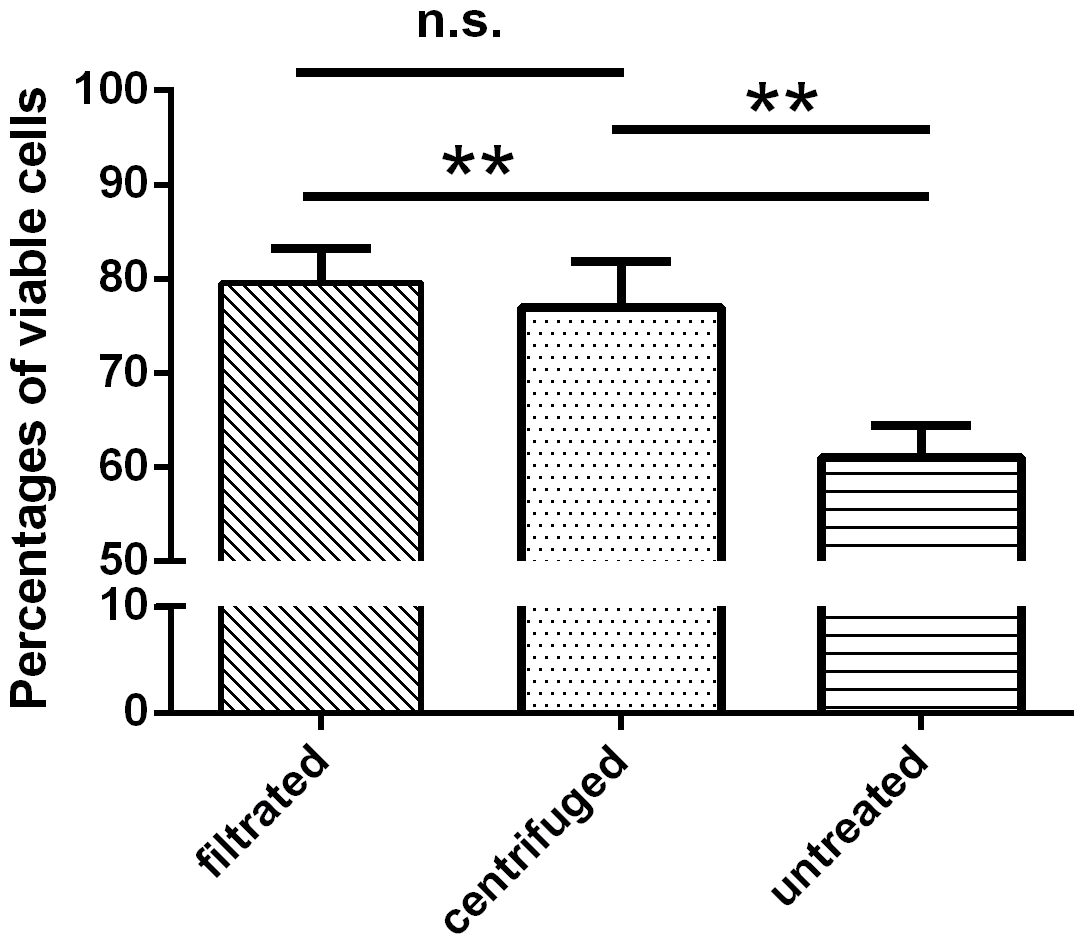
Percentages of viable *C. jejuni* DSM 4688^T^ determined by fluorescence microscopy before (untreated) and after filtration or centrifugation of cells. Results are shown as average from three independent experiments. Significant differences compared to untreated cells are indicated by asterisks (ANOVA, ** *p*<0.01, n.s. not significant).

The proportion of viable and nonviable *C. jejuni* in membrane filtrated, centrifuged, and untreated samples was investigated by fluorescence microscopy. For this, NaCl was spiked with 3.0×10^5^–1.5×10^6^ CFU/ml *Campylobacter* cells prior to recovery. Analysis of six fluorescence microscopy images from each of the three replicates revealed a significantly (*p*<0.05) lower mean percentage of viable bacteria (green) in untreated samples (61.08%) compared to filtrated (79.55%) or centrifuged (76.95%) samples, whereas the percentages of nonviable cells (red) were 38.92%, 20.45%, and 23.05%, respectively, in untreated, filtrated, and centrifuged samples (Figure 1). No intermediate (orange) cells could be detected, and the proportions of viable and nonviable cells did not differ significantly between filtrated and centrifuged samples (Figure 1).

### Impact of centrifugation and filtration methods on qPCR results with and without EMA

**Figure 2:**
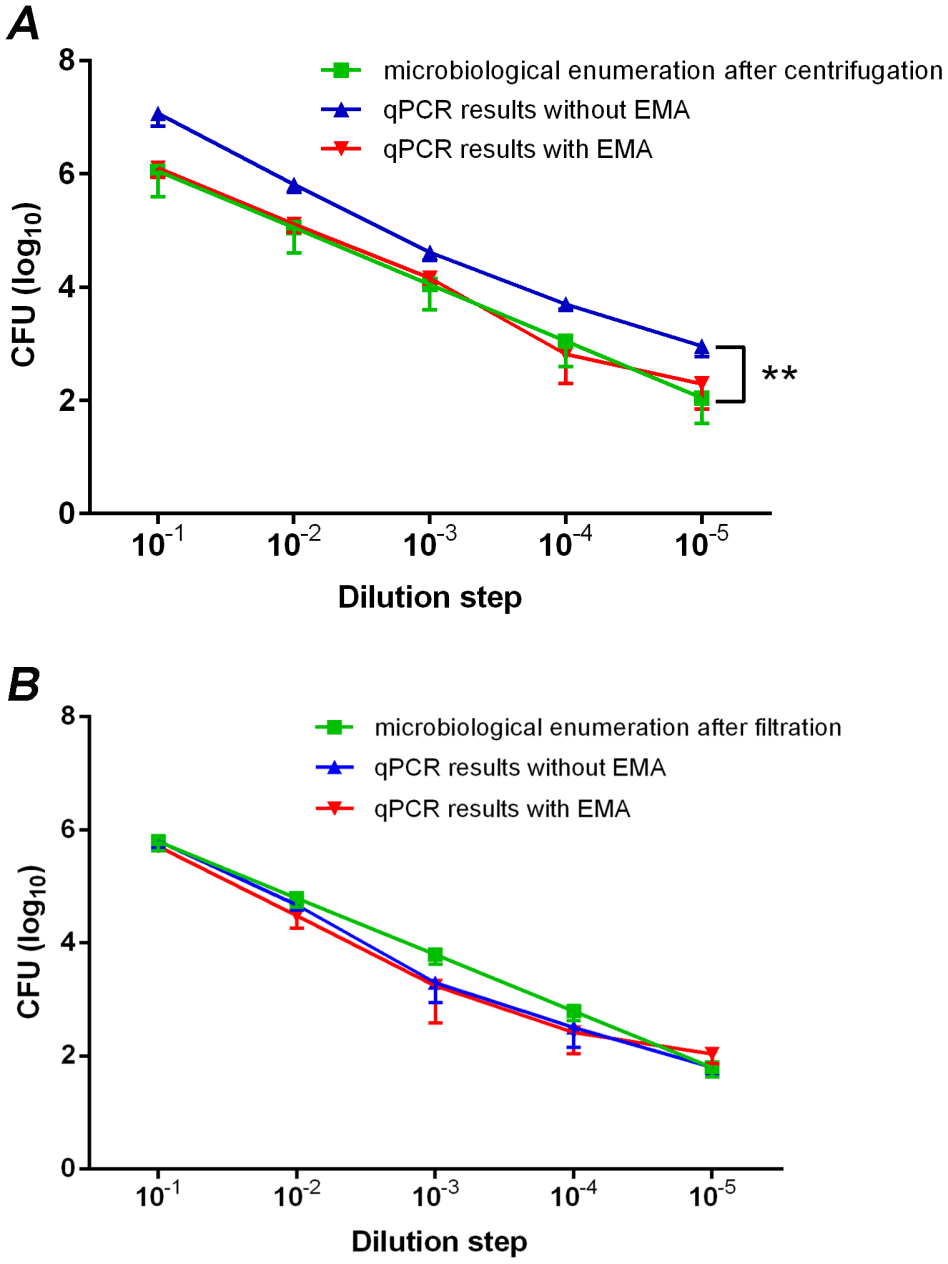
*Campylobacter* counts (CFU/ml) determined by culture enumeration, qPCR and EMA-qPCR after (A) centrifugation or (B) filtration of cells. Bacteria were recovered from NaCl suspensions starting from 3.0×10^5^–1.5×10^6^ *C. jejuni* DSM 4688^T^ cells/ml and diluted in a 10-fold dilution series. Results are shown as average from three independent experiments (ANOVA, ** *p*<0.01).

Following either the centrifugation or filtration of samples, the amounts of viable *C. jejuni* DSM 4688^T^ cells were quantified in parallel by qPCR and EMA-qPCR. For this, serial dilutions of cells suspended in NaCl were used, and results were compared to microbiological cell enumeration. Without the addition of EMA, qPCR-based quantification detected significantly (*p*<0.05) higher numbers of cells (+0.78±0.184 SD log_10_ CFU/ml) in the filtered samples compared to the microbiological reference method after centrifugation of samples (Figure 2A). However, EMA-qPCR results of the centrifuged samples were comparable (*p*>0.05) with microbiological cell counts. The Pearson correlation analysis was used to calculate the relationship between cell numbers determined by qPCR or EMA-qPCR, and microbiological cell counts; despite the shift of the curves, a high correlation between microbiological cell enumeration and qPCR results without EMA (R^2^ = 0.925) and with EMA (R^2^ = 0.866) was obtained (Figure S2).

In contrast, after membrane filtration of samples, qPCR-based quantification with and without pretreatment of EMA was comparable (*p*>0.05) to microbiological cell enumeration (Figure 2B). Again, high correlation coefficient values of R^2^ (0.942, slope 1.001; without EMA, and 0.893, slope 0.954; with EMA) were achieved (Figure S3). The results showed that the filtration method was more appropriate for the isolation of *Campylobacter* from water; therefore, this method was used for subsequent experiments.

### Comparison of the physicochemical and microbiological analyses of pond and tap water samples

The results from the physicochemical and microbiological analyses of pond and tap water samples are shown in Table 1. As was expected from the visual assessment, the turbidity of pond water was much higher (128.8 NTU) than that of tap water (0.17 NTU). This finding was also reflected in a more than 4-fold higher content of total organic carbon in the pond water sample. While the concentrations of most cations and anions were comparable, the microbiological analysis revealed a total viable count of bacteria of approximately 10^3^ CFU/ml and the presence of enterococci (67 CFU/100 ml) in the pond water sample. Contamination with *Campylobacter* spp. was detected in neither pond nor tap water samples.

**Table 1:** Results from the physicochemical and microbiological water analysis.

Parameter	Unit	Maximum permissible value^1^	Tap water^2^	Pond water
turbidity	NTU	1.0	0.17	128.8
pH	pH	6.5–9.5	7.68	7.6
electric conductivity (25°C)	µS/cm	2790	559	493
calcium	mg/l	-	71	82.8
potassium	mg/l	-	1.9	5.52
magnesium	mg/l	-	5.6	2.84
ammonium	mg/l	0.5	<0.07	0.087
iron	mg/l	0.2	<0.02	0.719
manganese	mg/l	0.05	0.01	0.174
nitrate	mg/l	50	2.7	<1
nitrite	mg/l	0.5	<0.01	<0.01
acid capacity up to pH = 4.3	mmol/l	-	2.31	2.8
total organic carbon (TOC)	mg/l	-	2.8	13.0
total hardness	°d	-	11.6	12.2
total viable count of bacteria (36°C/24 h)	cfu/ml	100	0	2.31×10^3^
total viable count of bacteria (20°C/44 h)	cfu/ml	100	0	3.31×10^3^
*Escherichia coli*	cfu/100 ml	0	0	0
Enterococci	cfu/100 ml	0	0	67
*Campylobacter* spp.	cfu/ml	-	0	0

1 values are obtained from the German drinking water regulation [37].

2 results are shown as mean values from January – December 2013.

### Influence of different types of water on the ratios of viable *Campylobacter* cells

**Figure 3:**
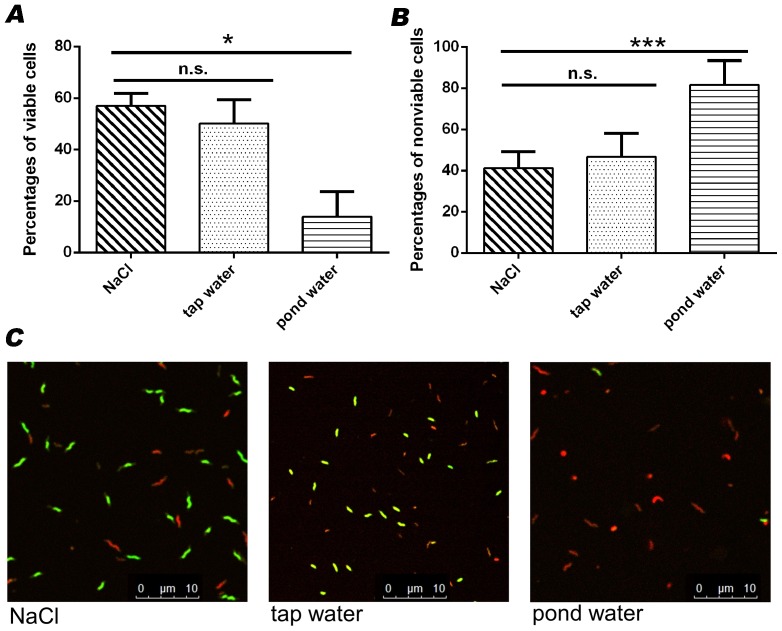
Percentages of (A) viable and (B) nonviable *C. jejuni* DSM 4688^T^ determined by fluorescence microscopy after inoculation of cells in NaCl, tap water and pond water. Percentages of intermediate cells were not included in the bar diagrams. Results are shown as average from three independent experiments. Significant differences compared to untreated cells are indicated by asterisks (ANOVA, * *p*<0.05, *** *p*<0.001, n.s. not significant). (C) Fluorescence images of cells after inoculation in NaCl, pond water or tap water. Green: viable bacteria; red: nonviable bacteria; orange: intermediate bacteria.

Fluorescence microscopy was used to determine proportions of viable and nonviable cells after inoculation of *Campylobacter* in pond or tap water. For this, water samples and control NaCl were spiked with approximately 5.0×10^5^ CFU/ml *C. jejuni* DSM 4688^T^ and incubated for 45 min at room temperature. Determination of the percentage of viable and dead cells revealed mean percentages of 50.18% viable and 46.73% nonviable cells in tap water; thus, these values were comparable to the results obtained in spiked NaCl samples (56.87% and 41.10%, respectively). No statistically significant differences (*p*>0.05) could be detected between the media. In contrast, inoculation of cells in pond water revealed percentages of 13.82% viable and 81.59% nonviable cells (Figure 3). The values of both parameters differed significantly (*p*<0.05) from the results of the control (0.9% NaCl) samples. Deviations from 100% resulted from the proportions of intermediate cells.

### qPCR and EMA-qPCR detection of *Campylobacter* cells from different types of water

**Figure 4:**
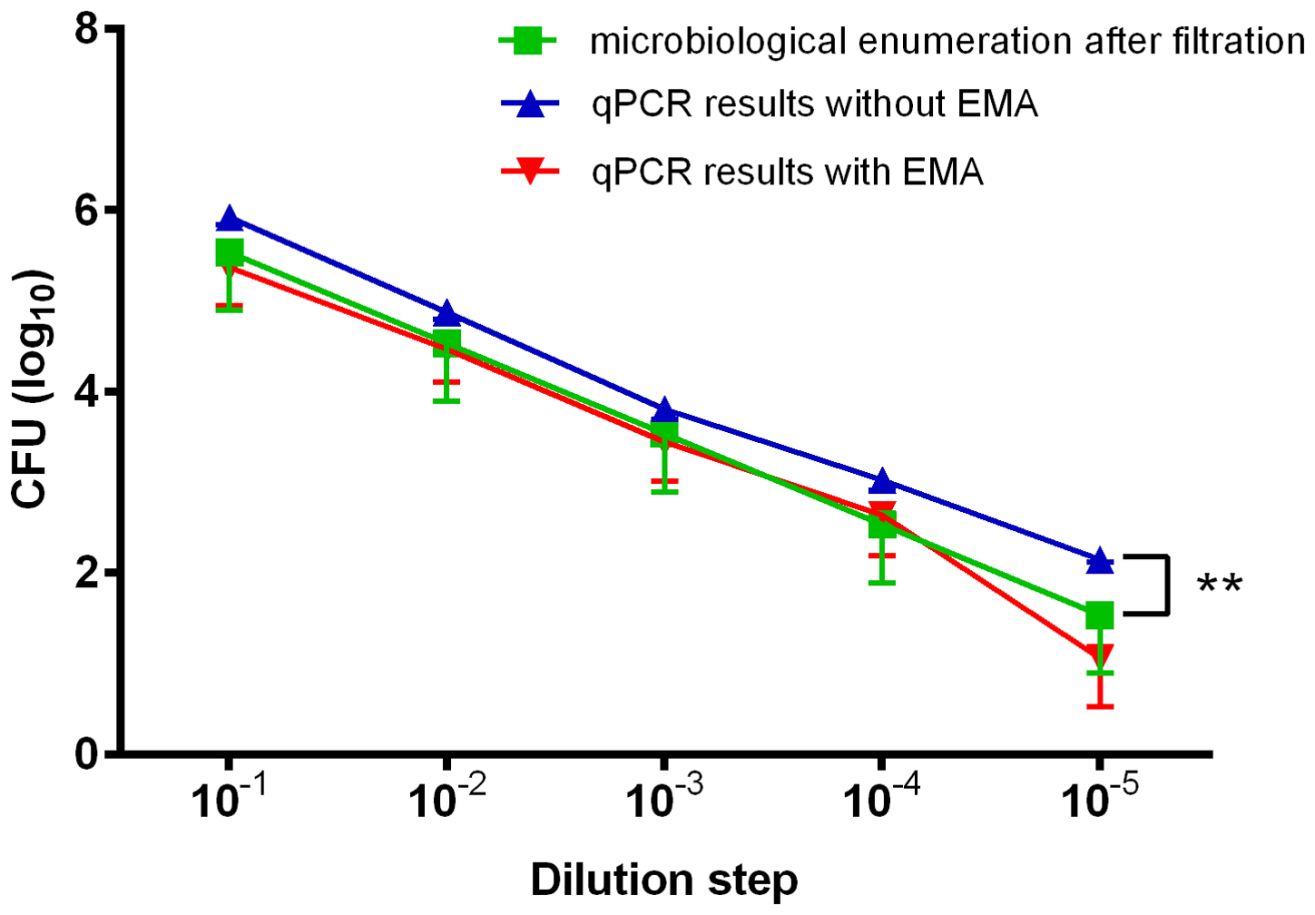
*Campylobacter* counts (CFU/ml) in tap water determined by bacteriological enumeration compared to results from qPCR and EMA-qPCR. Tap water was spiked with 3.0×10^5^–1.5×10^6^ *C. jejuni* DSM 4688^T^ cells/ml, diluted in a 10-fold dilution series and bacteria were recovered from water samples via membrane filtration. Results are shown as average from three independent experiments (ANOVA, ** *p*<0.01).

**Figure 5:**
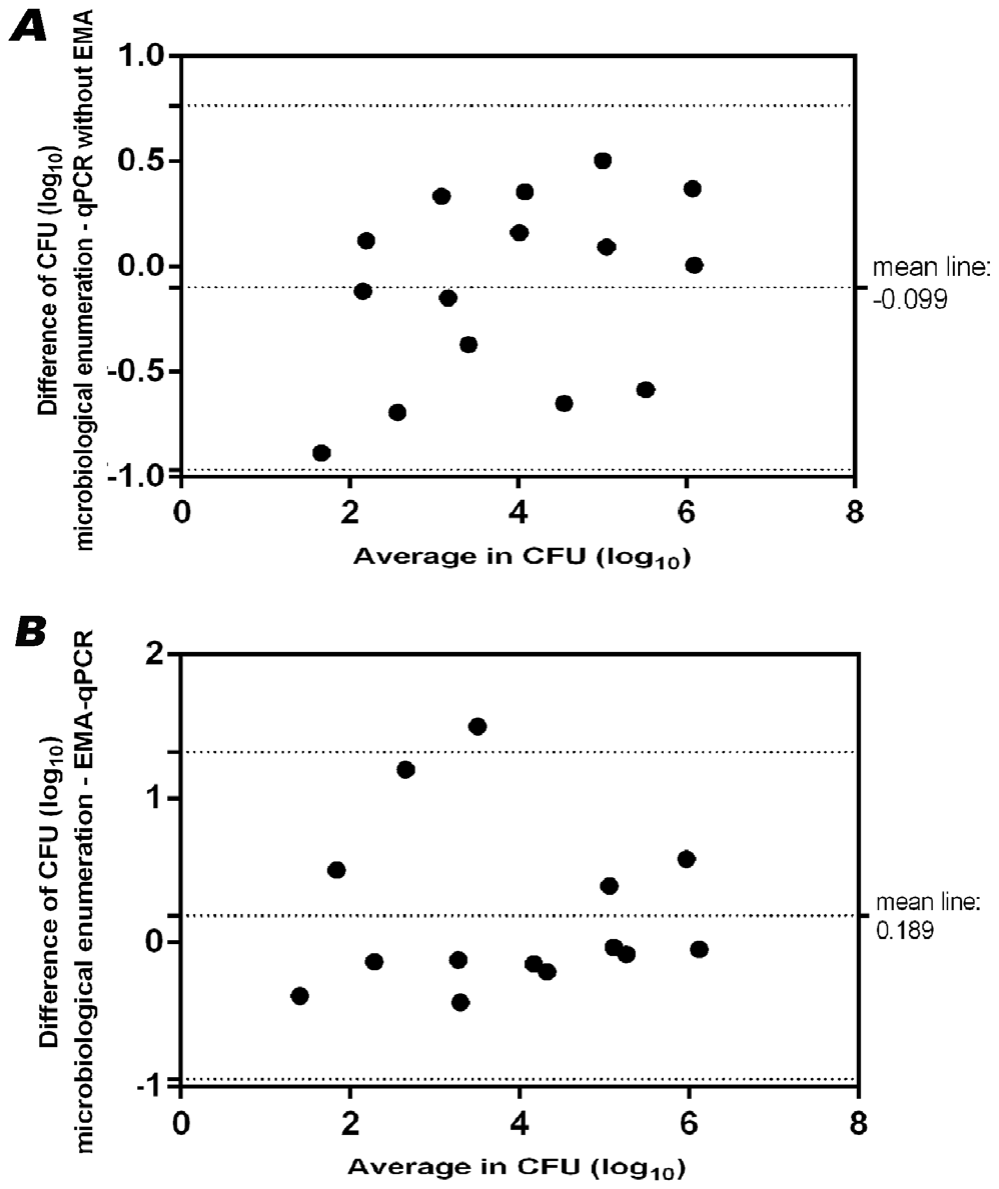
Bland-Altman plot for CFU (log_10_) determined by culture enumeration and qPCR results (A) without EMA and (B) with EMA. The middle line represents the mean difference of methods. Dotted lines above and below represent 95% limits of agreement.

**Figure 6:**
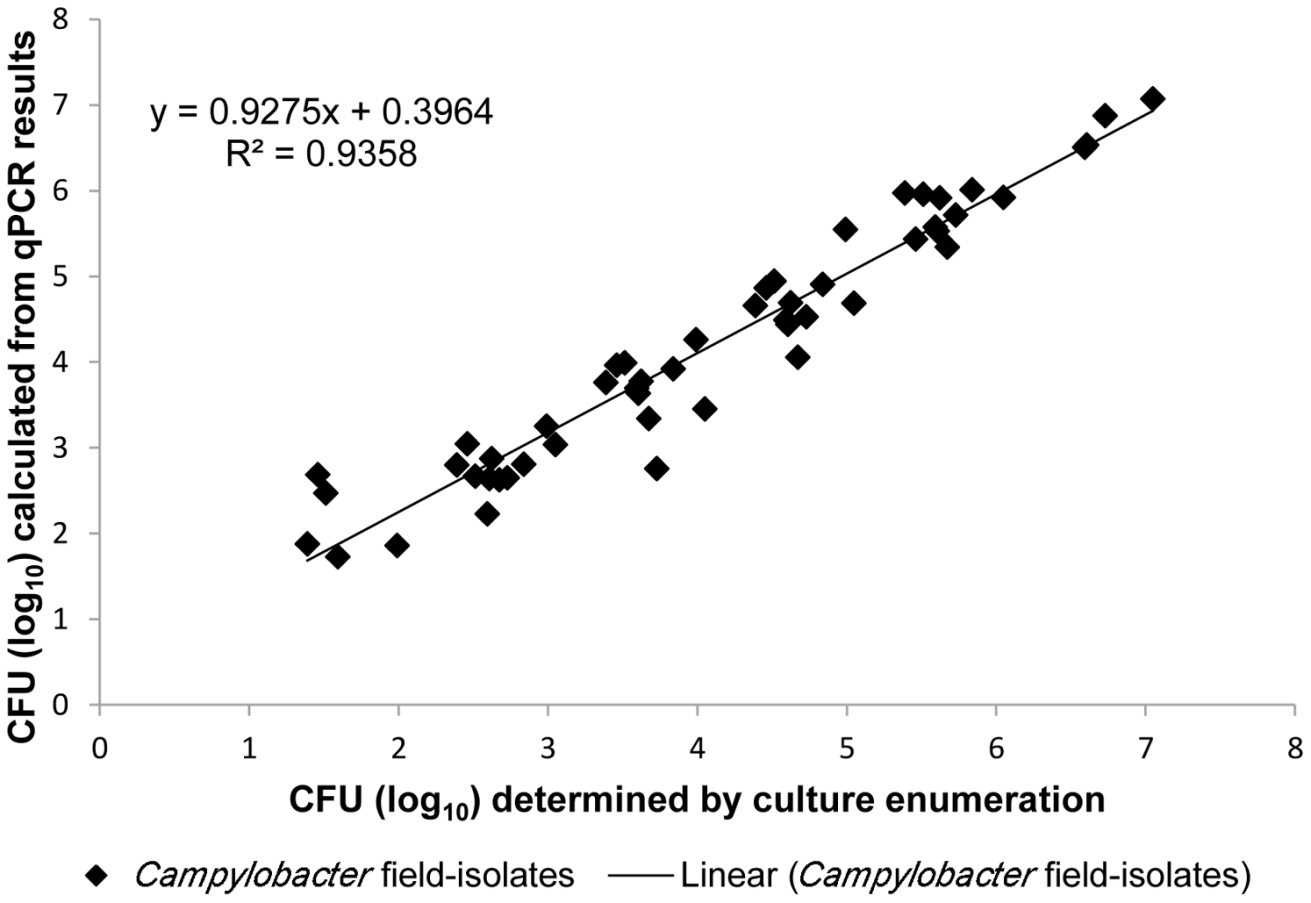
Scatter plot demonstrating the correlation between log_10_ numbers of CFU/ml *Campylobacter* field isolates calculated from EMA-qPCR results and the bacterial culture method.

The relationship between qPCR or EMA-qPCR results, and culture-based enumeration was assessed after recovery of *Campylobacter* cells from water samples. For this, tap water was spiked with 1.5×10^7^–2.3×10^2^ CFU/ml of reference strain *C. jejuni* DSM 4688^T^, and the amounts of cells were calculated after recovery by membrane filtration. Even though qPCR-based quantification of *Campylobacter* without EMA detected slightly higher (*p*<0.05) amounts of cells (mean difference: 0.42±0.133 SD log_10_ CFU/ml) than the microbiological reference method (Figure 4), the correlation coefficients of qPCR (R^2^ = 0.921; slope, 0.864) and EMA-qPCR (R^2^ = 0.863; slope, 0.939) versus culture enumeration were comparable. This finding was also reflected by Bland-Altman analysis. In the absence of EMA, the mean difference between the two methods was −0.099±0.442 SD log_10_ CFU and the 95% limit of agreement was 0.766 to −0.965 CFU (log_10_) (Figure 5A). Similarly, the Bland-Altman plot determined a high agreement between the results from EMA-qPCR and culture enumeration. The mean difference in log_10_ CFU was 0.189 (±0.579 SD) with lower and upper limits of −0.946 and 1.323, respectively (Figure 5B). For the sake of completeness, 13 field isolates were subjected to comparable EMA-qPCR quantification as described above. For two isolates, results were achieved from EMA-qPCR, but culture-based enumeration failed to detect *Campylobacter*. Therefore, these strains were excluded from the Pearson correlation analysis. For the remaining strains, there was a linear relationship between the results from both methods (R^2^ = 0.936, slope 0.928) (Figure 6).

As the results indicated a general applicability of EMA-qPCR, this method was used for analysis of spiked pond water, rain water, and mineral water samples. Similar to results from tap water samples, the results from rain water and mineral water samples were in good accordance (R^2^ = 0.929; slope, 0.951 and R^2^ = 0.989; slope, 1.013, respectively) with the reference method (Figure S4). Results from Bland-Altman plots also showed low variations between the methods. For rain water samples, the mean difference was 0.138±0.439 (SD) log_10_ CFU and the 95% limit of agreement was 0.998 to −0.722 CFU (log_10_), whereas for mineral water samples, the mean difference in log_10_ CFU was 0.153±0.171 (SD) with upper and lower limits of 0.488 to −0.183 CFU (log_10_) (Figure S5). However, when pond water was used, no correlation (R^2^ = 0.0005; slope, 0.0143) between results from real-time PCR signals and the reference method could be detected (Figure S6) and a low level of agreement was confirmed by Bland-Altman analysis (mean difference of 1.119±1.166 SD, 95% limits of agreement were 3.405 and −1.166, respectively).

### Detection of viable *Campylobacter* in mixed samples of viable and heat-inactivated cells

Finally, the applicability of the EMA-qPCR method was assessed in tap water samples spiked with defined ratios (1:10 and 1:100) of viable and heat-killed cells of type strain DSM 4688^T^, *C. jejuni* (n = 2) or *C. coli* (n = 2) field isolates. With the limitation that a relatively low number of samples (n = 14) was included, Bland-Altman analysis indicated a high agreement between EMA-qPCR results and microbiological cell counts. The mean difference was 0.006±0.531 SD CFU (log_10_) and the lower and upper limits were 1.047 and −1.035 CFU (log_10_) (Figure S7). In contrast, results from qPCR experiments without EMA differed noticeably from CFU determination (mean: −1.827±1.054 SD log_10_ CFU, 95% limits of agreement −3.892 and +0.238).

## Discussion

The consumption of contaminated poultry meat is not the only source of human campylobacteriosis outbreaks. *Campylobacter* spp. also occur in aquatic environments; thus, thousands of people have been exposed to contaminated drinking water in recent years [19], [21], [27]. As a consequence, reports have been published on waterborne *Campylobacter* outbreaks and gastroenteritis infections [21], [28]. However, these cases may still be underestimated due to a lack of testing and notification systems in many countries [21]. As it is difficult to isolate *Campylobacter* cells from water, which was confirmed by results from two field isolates in our study, PCR-based methods have been applied for the quantitative detection of genome copies [17], [27], [29]. In contrast, little is known about the applicability of a viability qPCR approach to quantify *Campylobacter* from water samples. Previous studies used pretreatment with intercalating dyes in initial experiments to investigate the survival of cells in freshwater [25] and to inhibit PCR signals from heat-killed *Campylobacter* in river water [21], [26].

In this study, we investigated two factors (the method to recover cells and the type of water sample) that may influence the membrane integrity of cells, with the aim to investigate the applicability of a *Campylobacter* EMA-qPCR approach for water analysis. First, two methods to concentrate *Campylobacter* from water samples were comparatively analysed. In samples spiked with high amounts of *C. jejuni*, both methods led to a mean cell loss of approximately 1 log_(10)_ CFU/ml. This loss may be attributed to the incomplete retention of the pathogen on microfilters due to its small size and morphology, as previously proposed [17], or to a loss of cells when discarding the supernatant after the centrifugation of samples [27]. In qPCR experiments, recovery rates may be enhanced by the use of a specific elution buffer containing 3% beef extract and a higher pH (pH = 9.0) [17]. However, the use of this modified buffer seems to be inappropriate for a bacterial viability assessment, as survival rates of *Campylobacter* decreased rapidly [17].

Increased proportions of viable cells after centrifugation and membrane filtration of samples compared to untreated (NaCl) controls indicated a loss, in particular, of nonviable cells. If membrane-compromised cells pass through the pores of the filter or are suspended in the supernatant following centrifugation, this could impact the number of nonviable cells after applying both concentration methods. Nevertheless, considering that the EMA-qPCR approach only detects viable cells, negative effects on microbiological counts could be excluded. Although the increase in the proportion of viable cells was comparable between centrifuged and filtrated samples, results from qPCR without EMA directly following centrifugation overestimated the quantities of *Campylobacter* cells compared to the reference method (mean difference of 0.78 log_10_ CFU/ml), whereas EMA-qPCR did not. In contrast, no significant differences between qPCR and EMA-qPCR results could be detected following filtration of samples. This difference may be attributed to the incomplete removal of free DNA after centrifugation of samples and, thus, can be considered a comparative advantage of the filtration method. Both PCR techniques achieved high correlation coefficients (R^2^ = 0.942 without EMA and R^2^ = 0.893 with EMA) between *Campylobacter* quantities, as determined by qPCR and microbiological cell counts, indicating a basic suitability of EMA-qPCR following the concentration of cells via membrane filtration.

Second, the influence of water types was investigated with regard to the EMA-qPCR approach. Fluorescence microscopy detected a significant higher proportion of nonviable cells after inoculation of *C. jejuni* cells in pond water, whereas the proportions in tap water and NaCl were comparable. This result could suggest that the microbial background in pond water, which could not be completely excluded in experiments even after sterile filtration, was responsible for the high percentage of nonviable cells (81.59%) [30]. One should bear in mind that the live/dead differentiation of intercalating dyes, such as EMA, is only based on the membrane integrity of cells, whereas penetration into the cell is not selective for a certain bacterial species [31]. As was expected from our findings and from previous publications [24], [32], [33], intercalating dyes are unable to sufficiently suppress signals from higher numbers of nonviable cells. Therefore, it is suggested that EMA-qPCR failed to detect the correct quantities of viable cells in pond water samples due to the high background of dead cells. In addition, a high turbidity of pond water was detected by physicochemical analyses, and these particles may be retained on microfilter membranes during filtration. As a consequence, these particles may be rinsed from the membranes together with bacteria, and may inhibit the photoactivation of EMA, which is necessary to induce binding of the intercalating dye to DNA. However, whether the combined pre-enrichment of samples and qPCR for quantification is an option for analysis of pond water samples, as recently proposed for *Salmonella* from pig carcasses, must be elucidated [34].

In contrast, investigation of spiked tap water samples revealed a general agreement between the methods. *Campylobacter* CFU calculated from qPCR and EMA-qPCR signals were approximately equivalent to those from microbiological cell counts, as shown by Pearson correlation analysis and a Bland-Altmann plot. For analysis of samples containing viable cells, it appears that both PCR techniques can be used interchangeably, whereas an advantage of EMA-qPCR could be demonstrated after analysing defined live/dead mixtures of *Campylobacter* cells up to a 1:100 dilution. Hence, these results are in good accordance with reports on *Pseudomonas aeruginosa* and *Legionella* [35], [36], in which qPCR techniques were successfully used with intercalating dyes to distinguish live from membrane-compromised cells after filter-based recovery of cells from water samples.

In conclusion, membrane filtration in combination with EMA-qPCR seems to be a promising method for the detection and differentiation of viable *Campylobacter* cells in tap water samples. However, further studies including an EMA-qPCR analysis of live/dead mixtures of bacterial cells and of higher numbers of field isolates are needed to validate the method.

## Supporting Information

Figure S1 **Microbiological cell enumeration before and after centrifugation or filtration of samples.**
(DOCX)

Figure S2 **Pearson-Regression analysis calculated from qPCR results and culture enumeration after centrifugation of samples.**
(DOCX)

Figure S3 **Pearson-Regression analysis calculated from qPCR results and culture enumeration after filtration of samples.**
(DOCX)

Figure S4 **Scatter plot demonstrating the correlation between culture enumeration and EMA-qPCR results.**
(DOCX)

Figure S5 **Bland-Altman plot for CFU determined by culture enumeration and qPCR results.**
(DOCX)

Figure S6 **Correlation between EMA-qPCR results and culture enumeration demonstrated by a scatter plot.**
(DOCX)

Figure S7 **Bland-Altman plot for CFU determined by culture enumeration and qPCR results.** Mixtures of viable and heat-killed *Campylobacter* cells were inoculated in tap water samples.
(DOCX)
