# Supplementary material for: Influencing Factors and Applicability of the Viability EMA-qPCR for a Detection and Quantification of Campylobacter Cells from Water Samples
Source: PLoS One. 2014 Nov 20;9(11):e113812. doi: 10.1371/journal.pone.0113812 (PMC4239115; doi:10.1371/journal.pone.0113812)
Supplement: Figure S7 — Bland-Altman plot for CFU determined by culture enumeration and qPCR results. Mixtures of viable and heat-killed Campylobacter cells were inoculated in tap water samples. (DOCX) [file pone.0113812.s007.docx]

**Without EMA**

**With EMA**
