# Supplementary material for: Influencing Factors and Applicability of the Viability EMA-qPCR for a Detection and Quantification of Campylobacter Cells from Water Samples
Source: PLoS One. 2014 Nov 20;9(11):e113812. doi: 10.1371/journal.pone.0113812 (PMC4239115; doi:10.1371/journal.pone.0113812)
Supplement: Supplementary file 5 [file pone.0113812.s002.docx]

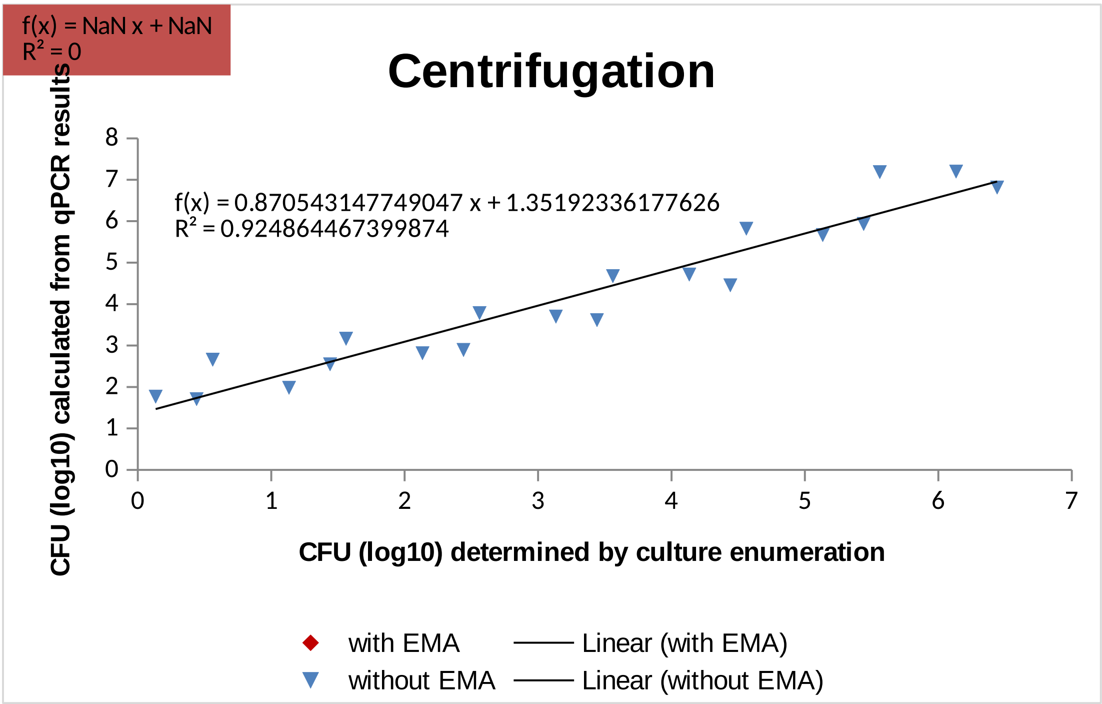

f(x) = NaN x + NaN
R² = 0
Centrifugation
CFU (log10) calculated from qPCR results
8
7
6
5
4
3
2
1
0
f(x) = 0.870543147749047 x + 1.35192336177626
R² = 0.924864467399874
0
1
2
3
4
5
6
7
CFU (log10) determined by culture enumeration
with EMA
without EMA
Linear (with EMA)
Linear (without EMA)
