# Supplementary material for: Influencing Factors and Applicability of the Viability EMA-qPCR for a Detection and Quantification of Campylobacter Cells from Water Samples
Source: PLoS One. 2014 Nov 20;9(11):e113812. doi: 10.1371/journal.pone.0113812 (PMC4239115; doi:10.1371/journal.pone.0113812)
Supplement: Supplementary file 6 [file pone.0113812.s003.docx]

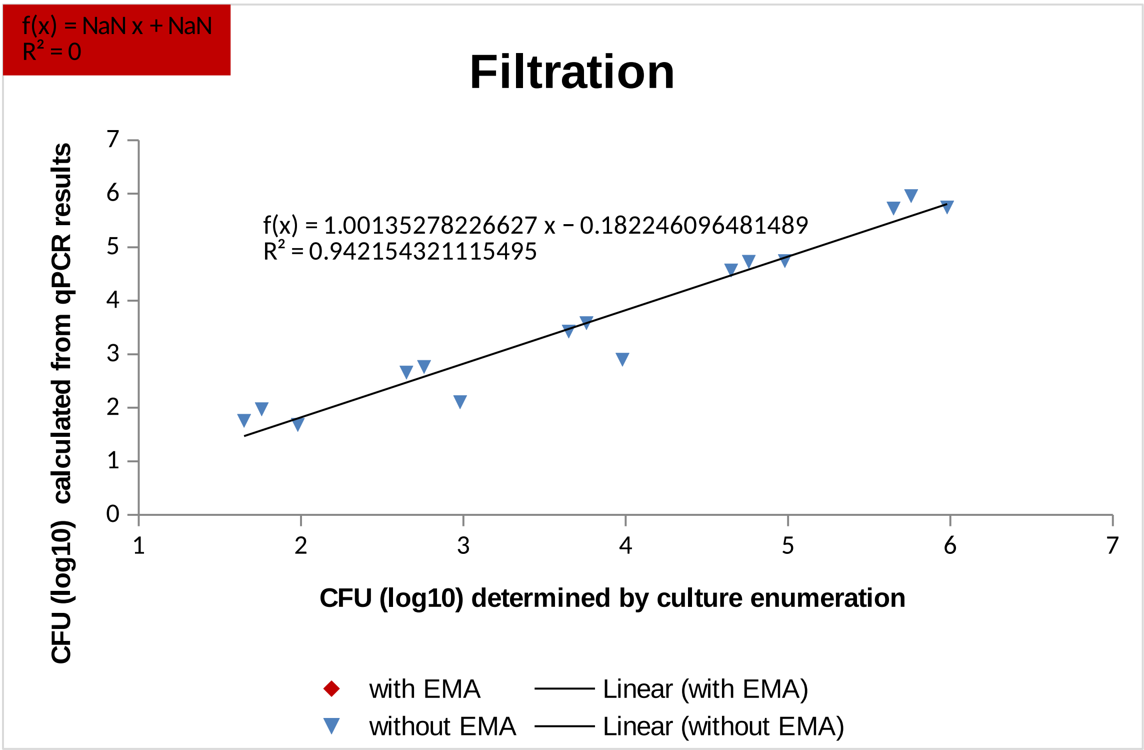
f(x) = NaN x + NaN
R² = 0
Filtration
f(x) = 1.00135278226627 x − 0.182246096481489
R² = 0.942154321115495
CFU (log10) calculated from qPCR results
CFU (log10) determined by culture enumeration
0
1
2
3
4
5
6
7
with EMA
without EMA
Linear (with EMA)
Linear (without EMA)
