# Supplementary material for: Influencing Factors and Applicability of the Viability EMA-qPCR for a Detection and Quantification of Campylobacter Cells from Water Samples
Source: PLoS One. 2014 Nov 20;9(11):e113812. doi: 10.1371/journal.pone.0113812 (PMC4239115; doi:10.1371/journal.pone.0113812)
Supplement: Supplementary file 7 [file pone.0113812.s004.docx]

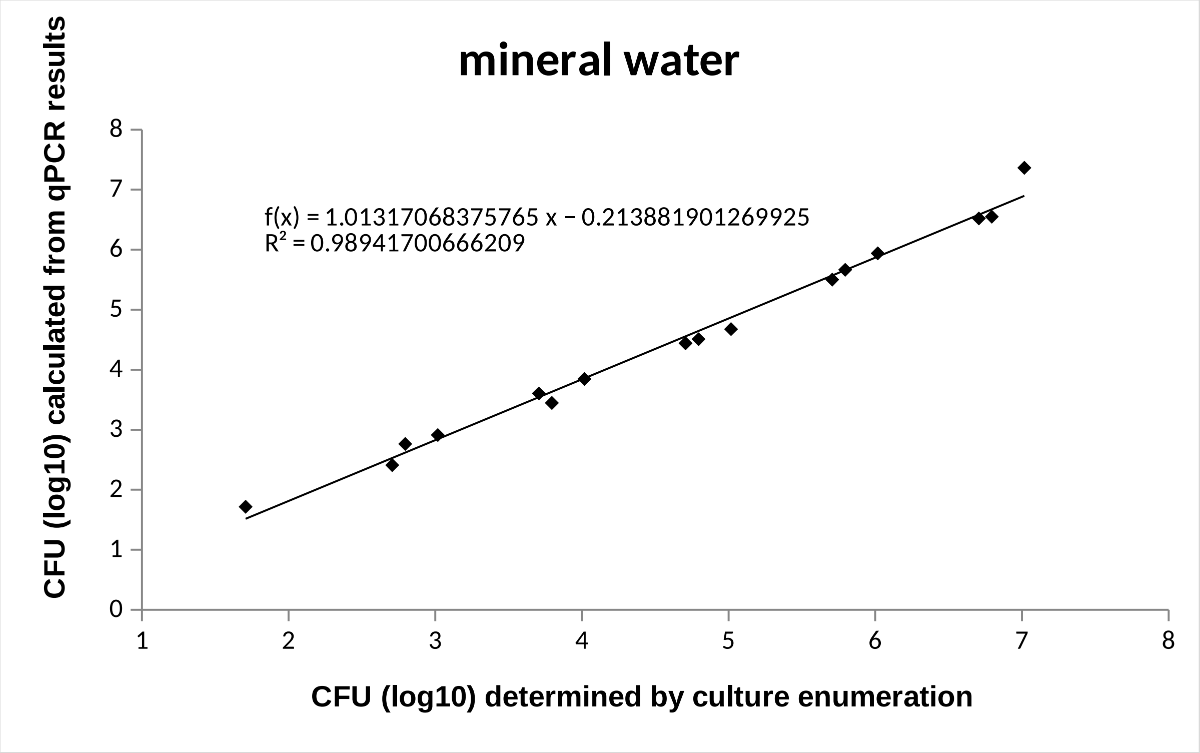
mineral water
f(x) = 1.01317068375765 x − 0.213881901269925
R² = 0.98941700666209
CFU (log10) calculated from qPCR results
CFU (log10) determined by culture enumeration
0
1
2
3
4
5
6
7
8
1
2
3
4
5
6
7
8

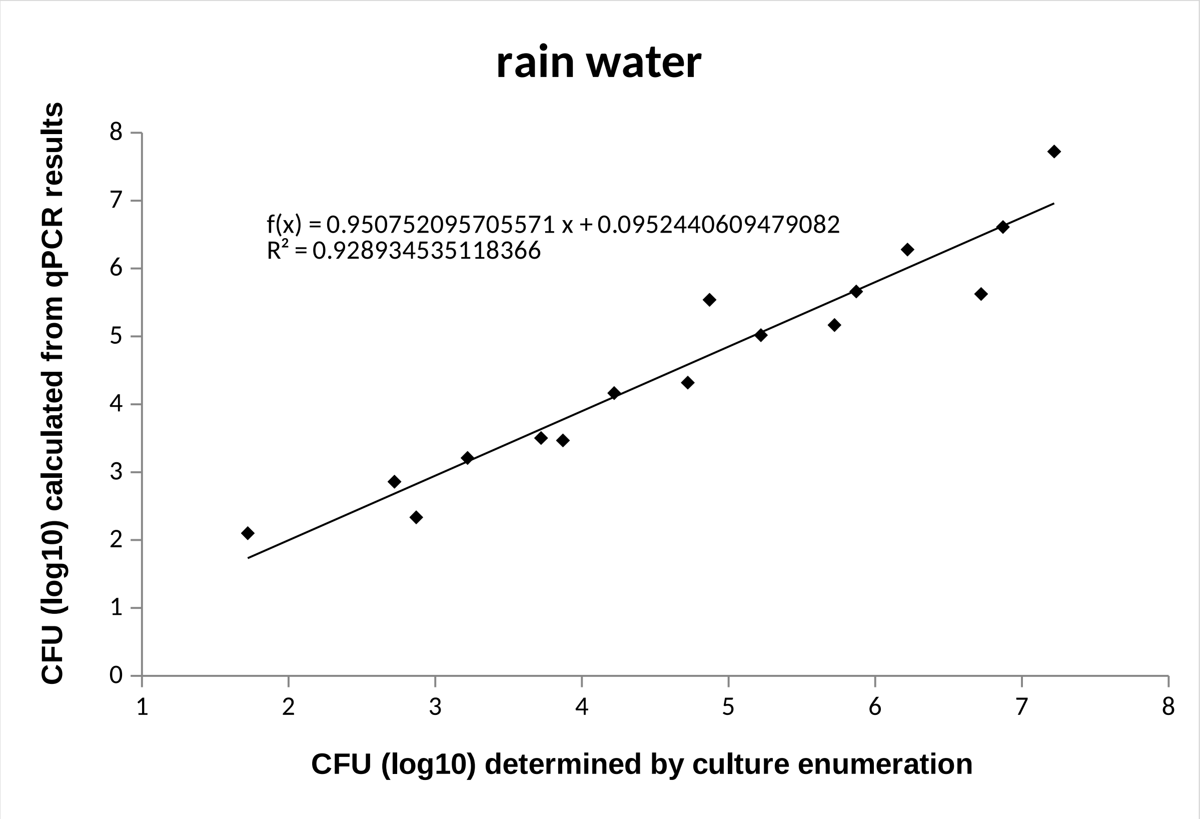
rain water
f(x) = 0.950752095705571 x + 0.0952440609479082
R² = 0.928934535118366
CFU (log10) calculated from qPCR results
CFU (log10) determined by culture enumeration
0
1
2
3
4
5
6
7
8
1
2
3
4
5
6
7
8
